# Glutathione S-Transferases Interact with AMP-Activated Protein Kinase: Evidence for S-Glutathionylation and Activation *In Vitro*

**DOI:** 10.1371/journal.pone.0062497

**Published:** 2013-05-31

**Authors:** Anna Klaus, Sarah Zorman, Alexandre Berthier, Cécile Polge, Sacnicte Ramirez, Sylvie Michelland, Michel Sève, Didier Vertommen, Mark Rider, Nicolas Lentze, Daniel Auerbach, Uwe Schlattner

**Affiliations:** 1 Université Grenoble Alpes, Laboratory of Fundamental and Applied Bioenergetics, Grenoble, France; 2 Inserm, Grenoble, France; 3 CRI-Inserm, Institut Albert Bonniot, Grenoble, France; 4 Centre Hospitalier Universitaire Grenoble, Plate-forme Protéomique Prométhée, Institut de Biologie et Pathologie, Grenoble, France; 5 Université Catholique de Louvain, Faculty of Medicine and de Duve Institute, Brussels, Belgium; 6 Dualsystems Biotech AG, Schlieren, Switzerland; University of Sassari, Italy

## Abstract

AMP-activated protein kinase (AMPK) is a cellular and whole body energy sensor with manifold functions in regulating energy homeostasis, cell morphology and proliferation in health and disease. Here we apply multiple, complementary *in vitro* and *in vivo* interaction assays to identify several isoforms of glutathione S-transferase (GST) as direct AMPK binding partners: Pi-family member rat GSTP1 and Mu-family members rat GSTM1, as well as *Schistosoma japonicum* GST. GST/AMPK interaction is direct and involves the N-terminal domain of the AMPK β-subunit. Complex formation of the mammalian GSTP1 and -M1 with AMPK leads to their enzymatic activation and in turn facilitates glutathionylation and activation of AMPK *in vitro*. GST-facilitated S-glutathionylation of AMPK may be involved in rapid, full activation of the kinase under mildly oxidative physiological conditions.

## Introduction

AMP-activated protein kinase (AMPK) is an evolutionary conserved heterotrimeric serine/threonine kinase that plays a central role in sensing and regulating energy homeostasis at the cellular, organ and whole-body level (recently reviewed in [1]–[7]). It exerts pleiotropic control of metabolic pathways and other physiological functions like cell growth, proliferation, motility or appetite control by affecting enzyme activities and transcription. This has made the kinase a prime pharmacological target for treating metabolic disorders or cancer [6], [8]. Activation of AMPK is triggered by a diverse array of external (e.g. hormones, cytokines, nutrients) and internal signals (e.g. AMP, ADP) linked to limited energy availability in physiological and pathological situations. Activation involves covalent phosphorylation of the α-subunit and allosteric binding of AMP or ADP to the γ-subunit. Covalent activation is complex, since it involves stimulated phosphorylation by upstream kinases (LKB1, CamKKβ) and inhibited dephosphorylation by phosphatases, both favored by binding of AMP and also ADP to different sites in the γ-subunit [9], [10] and myristoylation at the β-subunit [11].

Increasing evidence suggests that AMPK is also activated by reactive oxygen or nitrogen species (ROS, RNS), although the involved mechanisms are not entirely clear. The known inhibitory effect on mitochondrial ATP generation may simply increase cytosolic ADP/ATP and AMP/ATP ratios [12], but also other, non-canonical activation mechanisms are conceivable. We and others have reported for example that ROS/RNS, in particular peroxynitrite, may interfere with AMPK upstream signaling [13]–[15]. *Vice versa*, AMPK activation is involved in downstream redox regulation that can prolong cell survival [16] and induces expression of anti-oxidative proteins like superoxide dismutase (SOD), catalase or thioredoxin [17]–[19].

More recently, S-glutathionylation of Cys299 and Cys304 in the AMPK α-subunit via exposure to the strong oxidant H_2_O_2_ was reported to activate AMPK [20]. This reversible posttranslational protein modification can act as a functional switch like the well known protein phosphorylation and also protect thiol groups against further oxidation (reviewed in [21]–[23]). In case of AMPK, it probably causes activating conformational changes similar to those provoked by AMP-binding [24], [25]. Protein S-glutathionylation is often induced non-enzymatically upon expose to strong oxidants, in particular *in vitro* in combination with high glutathione levels [21]–[23], as shown for AMPK in presence of 200 μM H_2_O_2_ [20]. Intracellular levels of H_2_O_2_, e.g. in human fibroblasts, may at best reach the low nanomolar range [26], thus spontaneous S-glutathionylation *in vivo* would occur rather slowly and at low levels [21]. It may be more important in pathological, highly oxidative situations which change the cellular thiol redox state (ratio of reduced to oxidized glutathione, GSH/GSSG) and generate radical intermediates or oxidized cysteins. *In vivo*, protein glutathionylation is rather facilitated by specific enzymes that may constitute a dynamically regulated S-glutathionylation cycle [21], [22], [27]. Sulfiredoxins (SRx) and glutaredoxins (Grx) can act in protein deglutathionylation, and the latter enzyme also catalyzes the inverse reaction. More recently, isoforms of glutathione S-transferase (GST [28]), mainly GSTP1, were identified as catalysts of protein S-glutathionylation [28]–[32] confirming earlier models proposed by Townsend, Tew and colleagues (for recent reviews see [33]–[35]).

GSTs occur as a large superfamily of mitochondrial and cytosolic proteins. In mammals, there are seven classes of cytosolic GSTs, including the Alpha-, Mu-, and Pi-families [36], and lower eukaryotes express orthologs of these. A GST of the unicellular parasite *Schistosoma japonicum* belonging to the Mu-family is well known as the GST-tag used in fusion proteins to favor solubility and purification of proteins [37]. Historically, GSTs were characterized as class II detoxification enzymes that react glutathione with electrophilic compounds like by-products of oxidative stress and xenobiotics, thus facilitating their elimination from the cell (reviewed in [36], [38], [39]). However, some GSTs are now also emerging as ligands or modulators of signaling kinases like JNK, ASK1, PKC, PKA or EGFR, where either the interacting kinase or the GST is modified, functionally altered or relocated within the cell [40]–[44]. In particular GSTP1 was proposed to initiate a coordinated redox regulation of stress kinases to reduce cell death [40], [44].

While this kind of redox regulation relies exclusively on GST-protein interactions, the catalytic activity of GST is required for its role in protein glutathionylation. By hydrogen bonding of glutathione to their active site tyrosine, GST-Alpha, -Mu and -Pi enzymes decrease the pKa of the glutathione thiol group (R-SH) and thus favor thiol deprotonation to form the highly nucleophilic thiolate anion (R-S^−^) [22], [45], [46]. Such activated glutathione is used in various detoxification reactions, but also allows for S-glutathionylation of sulfenic acids (-SOH) on proteins with low pKa cysteines [28], [29], [31]. Inversely, it has been speculated that low GST peroxidatic activity in presence of peroxides could generate glutathione sulfenic acid intermediates that would react with protein cysteine thiolates [22]. The exact biochemical determinants of GST-catalyzed S-glutathionylation remain to be fully established. In particular, most studies so far were dedicated to the role of GSTP1 following expose to high concentrations of ROS or RNS, while few is known on its role under more physiological conditions and other GST isoforms [21].

Here we describe an *in vitro* glutathionylation and activation of AMPK that is catalyzed by two mammalian GST isoforms, GSTM1 and -P1, and relies on close and direct interaction of these GSTs with the AMPK β-subunit as evidenced by multiple assays. Such AMPK/GST complexes may amplify kinase activation under mildly oxidative, physiological conditions.

## Materials and Methods

### Cloning and protein production

Cloning, expression and purification of GSTM1, GSTP1, GST-Sj, CamKKβ, AMPK α2β2γ1 (221WT) and AMPK α2T172Dβ2γ1 mutant (221TD) is described in [7], [47] and Methods S1. For phosphorylation assays, the N-terminal Strep-tag in GSTM1 and P1 was removed, since mass spectrometry identified a serine being phosphorylated by AMPK within this tag (peptide ASWpSHPQFEK, see Methods S1, Fig. S1).

### Yeast two-hybrid assays

Cytosolic yeast two-hybrid (Y2H) systems, Cyto-Y2H [48] and Split-Trp-Y2H [49], both as variants developed by Dualsystems Biotech (Schlieren, Switzerland), are described in Methods S1 and [48]. In short, GST and AMPK subunits were expressed as fusion proteins, in Cyto-Y2H with a membrane anchor and the C-terminal end of ubiquitin conjugated to a transcription factor (bait) or with the N-terminal end of ubiquitin (prey), and in Split-Trp-Y2H with the C-terminal (bait) or the N-terminal (prey) portion of Trp1p. Selective media to control the presence of bait and prey plasmid lacked tryptophan and leucine (SD-WL, Cyto-Y2H) or uracil and leucine (SD-UL, Split-Trp-Y2H), and additionally adenine and histidine (SD-AHWL, Cyto-Y2H) or tryptophan (SD-UWL, Split-Trp-Y2H) for protein interaction analysis. Spotted plates were incubated 72 h at 30°C (Cyto-Y2H) or up to 9 days at 27°C (Split-Trp-Y2H).

### Rat liver extracts, protein

Rat liver was obtained from animals anesthetized with sodium pentobarbital (40 mg/kg, i.p.) according to the protocol approved by the Grenoble Ethics Committee for Animal Experimentation (no. 36_LBFA-LK-01). Liver tissue was immediately extracted in 10 mM HEPES pH 7.4 (containing 220 mM mannitol, 70 mM sucrose, 0,1% bovine serum albumin (BSA), 0.2 mM EDTA) and centrifuged twice (1000 g and 12 000 g for 10 min each) to obtain soluble proteins in the supernatant for pull-down and immunoprecipitation. Protein concentrations were determined according to Bradford [50] with the Biorad microassay (Biorad, Reinach, Switzerland) and BSA as standard.

### GST pull-down, immunoprecipitation and immunoblotting

For pull-down assays, either 30 µg of purified recombinant protein (GST-Sj, rat GSTM1 and -P1, or GST-tagged acetyl-CoA carboxylase, GST-ACC [51]) or 1 mg proteins from liver extract were incubated with 30 µg recombinant AMPK (α2β2γ1 wild-type, 221WT; or T172Dα2β2γ1 mutant, 221TD) for 1 h in PD-buffer (20 mM HEPES pH 7.4, 50 mM NaCl, 2,5 mM MgCl_2_, 10% glycerol, 6 g/L BSA, 0,5% Tween 20, 0,02% NaN_3_) before addition of Glutathione Sepharose beads and incubation for an additional hour at 4°C. Where indicated, 1 mM glutathione was included. Sepharose beads were washed eight times and resuspended in SDS sample buffer. For immuno precipitation, 1 mg protein from liver extracts were reacted with anti-GSTM (ab53942, Abcam) or GSTP1 (ab53943, Abcam) antibody (1∶240) in PD-buffer overnight at 4°C. Protein A Sepharose was added, incubated for another hour at 4°C, and washed 8 times before being resuspended in SDS-PAGE sample buffer. Solubilized, denaturated proteins were subjected to SDS-PAGE and immunoblotting using anti-AMPKα primary antibody (dilution 1: 1000, 2532, Cell Signaling Technology, Danvers, MA, USA) and anti-rabbit secondary antibody (1: 5000, NA934, GE Healthcare) for detection with a chemiluminescence kit (ECL plus, GE Healthcare) and a CCD camera (ImagerQuant LAS 4000, GE Healthcare). Bands were quantified densitometrically by Image J (imagej.nih.gov/ij) and normalized. Statistical analysis was done by students T-test. Where indicated, proteins stained in PAGE gels with colloidal Coomassie Blue were identified by MALDI-TOF/TOF mass spectrometry.

### Surface Plasmon Resonance (SPR) and mass spectrometry (MS)

For SPR with BIAcore (GE Healthcare), GSTs were covalently immobilized by standard amine coupling (GE Healthcare) on the carboxylic functions of two different chips. Gold chips functionalized by mixed self-assembled monolayers as described [52] were kindly provided by Wilfrid Boireau (FEMTO-ST, CNRS Besançon, France). They used 97% 11-mercapto-1-undecanol to reduce non-specific adsorption of proteins to the surface, and 3% 16-mercaptohexadecanoic acid for protein immobilization to obtain well controlled, low ligand densities as convenient for initial experiments with GST-Sj. CM5 chips (GE Healthcare) allowing higher ligand surface densities were used for sequential analyte injection during detailed kinetic analysis. GST (30 µg/ml) in 10 mM acetate buffer pH 6 (GST-Sj), pH 5 (GSTM1) or pH 4 (GSTP1) were injected at 5 µl/min to immobilize ≈2.5 ng GST/mm^2^. Interaction measurements were carried out in running buffer (10 mM HEPES pH 7.4, 100 mM NaCl, 50 μM EDTA, 0.005% Surfactant P20) at a flow rate of 20 µl/min. AMPK diluted to different concentrations just prior to measurements was injected onto the GST surfaces for 180 to 300 s at 20 or 30 µl/min which excludes mass transfer limitations (not shown). Experimental curves were corrected for bulk refractive index changes. Fitting of association and dissociation curves for kinetic analysis was done with BIAevaluation software. GSTs were identified by MALDI-TOF/TOF and peptide mass fingerprinting, potential phosphosites by LC-MS/MS as described in Methods S1.

### AMPK glutathionylation

AMPK 221WT (1 μM, stocks preserved at −80°C) in 0.1 M phosphate buffer pH 6.5 was incubated with or without 10 mM glutathione alone or together with GSTM1 or -P1 (0.5 μM) for 10 min at 30°C. Alternatively, AMPK (1 μM, reduced by overnight incubation with 1 mM ß-mercaptoethanol in phosphate buffer as above) was incubated with EDTA (1 mM) at 30°C alone or together with GSTM1 or -P1 (10 μM, added after 5 min). After 15 min, 0,1 mM glutathione was added for 2 or 4 min. The reaction was stopped by heating in SDS sample buffer and samples separated by non-reducing SDS-PAGE and immunoblotted using primary anti-glutathione antibody (1∶1000, MAB5310, Millipore Corporation, Billerica, USA) and anti-mouse secondary antibody (1: 4000, 31430, Pierce, Rockford, USA) for luminescent detection and quantification as described above. Blotted proteins were also visualized by Ponceau staining to reveal M_r_ shifts due to glutathionylation.

### AMPK phosphorylation

AMPK 221WT (25 nM) was incubated for 10 min at 30°C with or without glutathione (10 mM) and in presence or absence of GSTM1 or -P1 (125 nM) in kinase buffer containing 200 μM [γ-^32^P]ATP (specific activity 400 mCi/mmol ATP), 50 μM AMP, 5 mM MgCl_2_, 1 mM DTT, and 10 mM HEPES (pH 7.4). Recombinant CamKKβ (1.25 nM) was added and samples were incubated for 3 min at 30°C. The reaction was stopped by heating in SDS sample buffer and AMPK phosphorylation at Thr-172 as an indicator of AMPK activity was probed by SDS-PAGE and immunoblotting with anti-phospho-T172 AMPKα primary antibody (1: 1000, 2531, Cell Signaling Technology, Danvers, MA, USA) and anti-rabbit secondary antibody for luminescent detection and quantification as described above.

### AMPK substrate phosphorylation

To analyze GST phosphorylation *in vitro*, AMPK 221WT (4 pmol) was activated by incubation with CamKKβ (1 pmol) for 20 min at 30°C in kinase buffer with cold ATP. Purified GSTs and ACC [51] (200 pmol each) were then incubated for 3–60 min at 37°C in the presence or absence of pre-activated AMPK 221WT (4 pmol) in kinase buffer. For negative controls, GSTs were incubated with 1 pmol CamKKβ alone without AMPK. To analyze effects of GST/AMPK complexes on *in vitro* phosphorylation of AMPK substrates, AMPK 221WT (4 pmol, reduced as above) was pre-activated with CamKKβ in kinase buffer with cold ATP and glutathionylated with 0,1 mM glutathione in presence or absence of GSTM1 or -P1, both as described above. Then, ACC (200 pmol) [51] and [γ-^32^P]ATP were added and the mixture incubated for 2 min at 37°C. Kinase reactions were stopped as above, separated on SDS-PAGE and analyzed by Typhoon phosphoimager (GE Healthcare).

## Results

### GST-Mu and -Pi isoforms interact with AMPK *in vitro*

**Figure 1:**
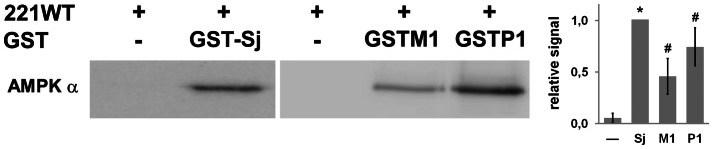
GST isoforms and GST-tag interact with full-length AMPK in pull-down assays. Pull-down of recombinant AMPK 221WT with GST-Sj (*S. japonicum*), GSTM1 or GSTP1 (*R. norvegicus*). In all assays, AMPK (0.075 mg/ml) was incubated with or without (negative control) GST proteins (0.075 mg/ml). Pull-down with Glutathione Sepharose 4B was subjected to immunoblot analysis using anti-AMPKα antibody. Left: representative data; right: quantification (mean ± SD, n = 3; * p<0,01 and ^#^ p<0,05 *versus* no GST).

In the course of our interactomic research on AMPK, we repeatedly pulled down recombinant heterotrimeric AMPK with recombinant proteins fused to a GST-tag that is derived from *S. japonicum* GST (GST-Sj). In fact, GST-Sj alone can pull down AMPK 221WT (Fig. 1). We first examined whether such heterologous interaction of GST-Sj with rat AMPK reflects an interaction that evolved with homologous rat GSTM1 (closest homologue of GST-Sj, 44% sequence identity) and GSTP1 (30% sequence identity). Both enzymes were cloned from a rat cDNA library, bacterially expressed and purified. In an assay with five fold molar excess of GST, both GSTM1 and –P1 were able to pull down AMPK 221WT even after extensive washing (Fig. 1). These results suggest that the GST/AMPK interaction evolved in at least two different eukaryotic GST classes: the Mu and Pi families.

### GST-Mu and -Pi isoforms directly interact with AMPK β-subunits in Y2H assays

A potential direct interaction of GSTs with AMPK *in vivo* was verified by two different last-generation Y2H assays [53]. Here, bait/prey interaction leads to reconstitution of split proteins in the yeast cytosol, either of ubiquitin (Cyto-Y2H) or an enzyme in tryptophan biosynthesis (Split-Trp-Y2H). Readout is provided via transcription factor release by ubiquitin-specific proteases that triggers transcription of reporter genes (Cyto-Y2H), or more directly by allowing growth on Trp-deficient medium (Split-Trp-Y2H) [49]. While the transcriptional amplification of the Cyto-Y2H read-out makes it very sensitive to detect even weak or transient interactions, the direct readout of the Split-Trp-Y2H is more proportional to interaction strength.

**Figure 2:**
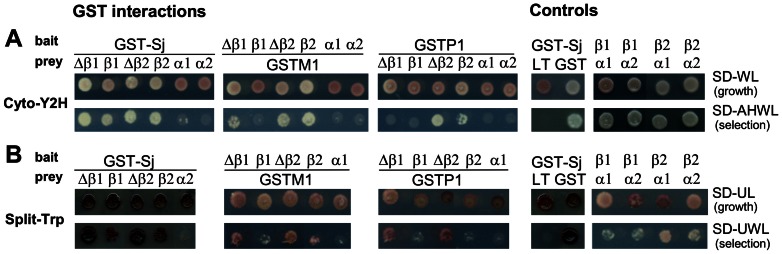
Y2H analysis identifies GST isoforms as AMPK interaction partners and the AMPK interaction domain. Two different cytosolic Y2H systems were applied to analyze interaction of AMPK with *Schistosoma japonicum* GST (GST-Sj) and mammalian (rat) GSTM1 and GSTP1. (A) Cyto-Y2H: interacting proteins lead to reconstitution of ubiquitin and a transcriptional readout allowing growth on medium lacking adenine and histidine (SD-AHWL). Spots represent yeast grown for 72 h at 30°C. (B) Split-Trp-Y2H: interacting proteins lead to reconstitution of Trp1p, an enzyme in tryptophan biosynthesis, and allow growth on medium lacking tryptophan (SD-UWL). Yeast was grown for 8–9 days at 27°C. Δβ, N-terminal domain of AMPK β-subunit. Controls: LT, Large T Antigen of *Simian Virus* (amino acids 84–704; negative control); GST, GST-Sj (positive control). A representative data set out of three independent experiments is shown. For more details see Materials and Methods and Supporting Information.

The three examined GSTs (GST-Sj, GSTM1, GSTP1) did not interact with AMPK α -subunits in the sensitive Cyto-Y2H assay (Fig. 2A). However, all three showed interaction with AMPK β-subunits: β1 and β2 in case of GST-Sj, and preferentially β2 in case of GSTM1 and -P1. The N-terminal domain of the β-subunits (Δβ1 or Δβ2, amino acids 1–54) was sufficient for GST/AMPK binding, suggesting that it is part of the interaction domain. Control experiments confirmed expected GST homodimerization and AMPK α/β-subunit interaction, while no binding to unrelated protein Large T (LT) was detected. The Split-Trp-Y2H assay confirmed these data (Fig. 2B), although the readout was weaker in some cases, as e.g. in case of the AMPK α-/β-subunit interaction. In both assays, results were similar, irrespective whether GST-Sj was used as bait or prey (not shown).

### GST/AMPK interaction occurs in rat liver

**Figure 3:**
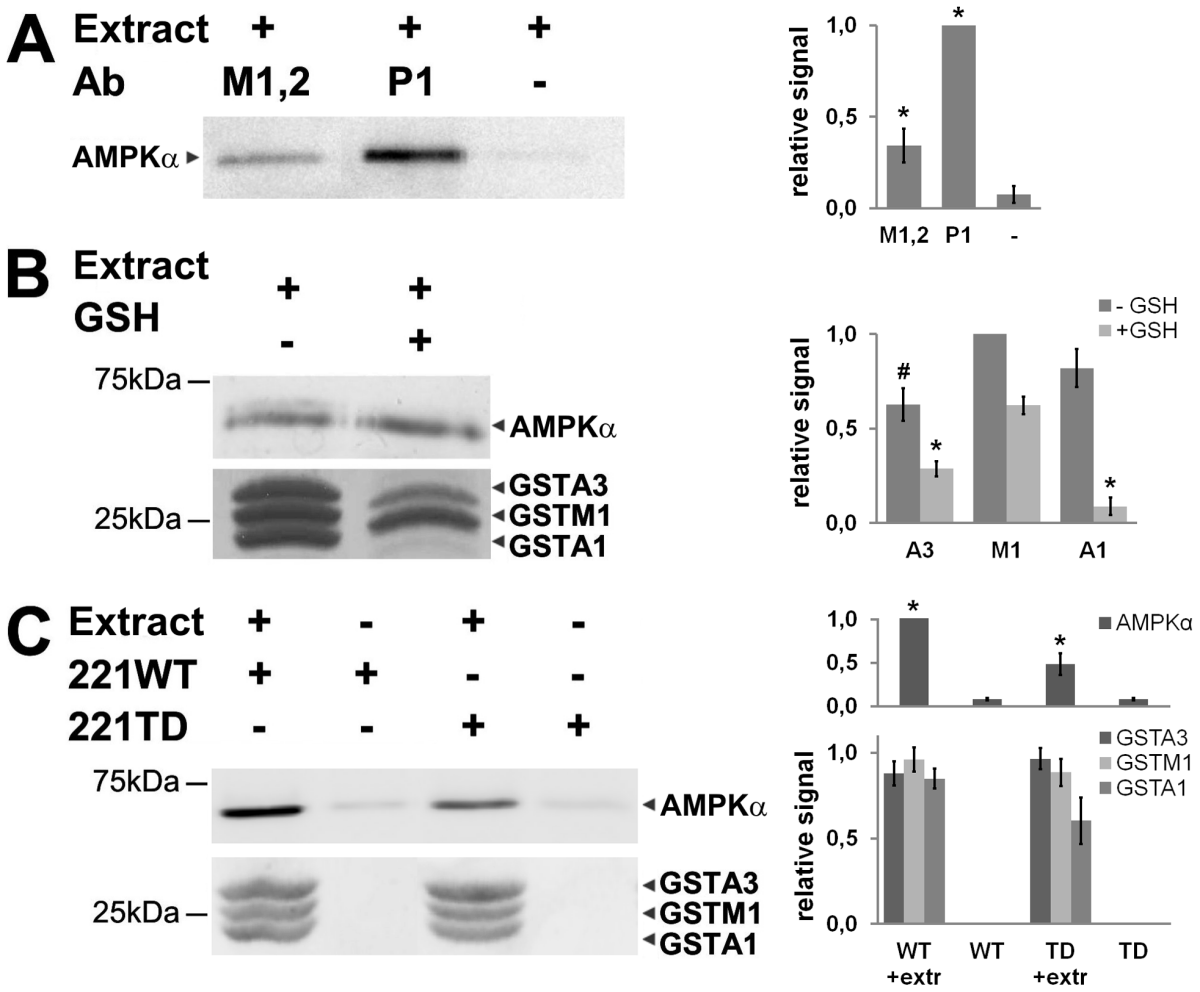
AMPK interacts with endogenous GST isoforms in rat liver. GST immunoprecipitation (A) or pull-downs (B, C) were performed with rat liver extract. AMPK in immunoprecipitates or pull-down fractions was detected by immunoblot analysis with anti-αAMPK antibody. The main liver GST isoforms in pull-down fractions were detected by Ponceau staining and mass spectroscopy. (A) Immunoprecipitation of endogenous AMPK by anti-GSTM1/2 or anti-GSTP1 antibodies. (B) GST pull-down of endogenous liver AMPK by liver GST isoforms in absence or presence of glutathione. Note: Addition of glutathione reduces pull-down of GSTA isoforms without affecting pull-down of AMPK. (C) GST pull-down of added AMPK 221WT or constitutively active 221TD. Left: representative data sets; right: quantification (mean ± SD, n = 3; * p<0,01 and ^#^ p<0,05 *versus* no GST (A), GSTM1 (B) or no extract (C)). Extr, liver extract.

To test whether also endogenous rat GST isoforms bind to AMPK, we used crude rat liver extracts for co-immunoprecipitation and GST pull-down assays. Liver contains mainly GST-Alpha and -Mu isoforms and few GST-Pi. Endogenous rat AMPK indeed co-immunoprecipitated with antibodies specific for GSTM1/2 and GSTP1 (Fig. 3A) and pulled down together with three major endogenous GST isoforms, GSTA1, GSTA3 and GSTM1, as identified by MALDI mass spectrometry (Fig. 3B). If glutathione is then added to the extract, the pull-down assay becomes more stringent. Mainly GSTM1 is now pulled down, while GSTA1 and GSTA3 are strongly reduced, without affecting the quantity of associated AMPK. Finally, when liver extracts were spiked with additional recombinant AMPK 221WT or 221TD, even more AMPK was pulled down (Fig. 3C). More inactive AMPK 221WT was recovered as compared to AMPK 221TD, a mutant mimicking active AMPK [54].

### GST/AMPK interaction is direct and rapid

**Figure 4:**
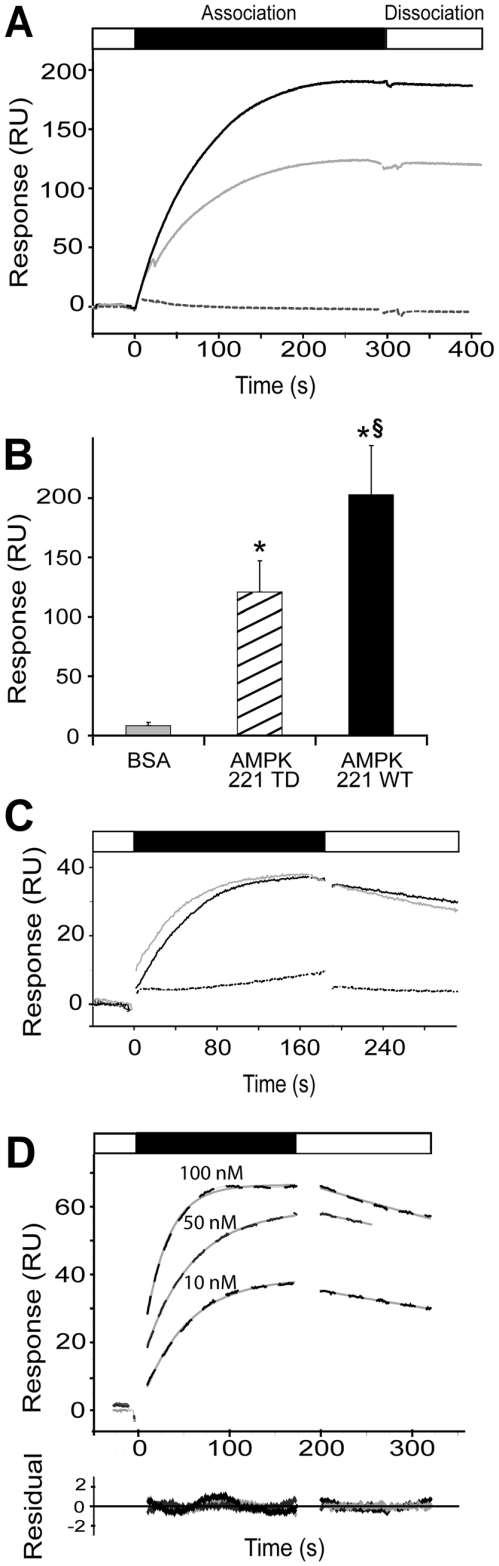
Surface plasmon resonance identifies high affinity interactions between GSTs and AMPK. Freshly diluted, recombinant full-length AMPK was injected onto immobilized GST. (A) GST-Sj binding of 10 nM AMPK 221WT (black full line), constitutive active AMPK 221TD (grey full line) or BSA (grey dotted line) at a flow rate of 20 µl/min (surface: self-assembled monolayer). (B) Equilibrium response from (A), mean ± SD, 12 (221TD), 6 (221WT) or 3 (BSA) independent experiments (* p<0,01 *versus* control; ^§^ p<0,01 *versus* AMPK-TD). (C) Comparison of GSTM1 (black) or GSTP1 (grey) association and dissociation kinetics of 10 nM AMPK 221WT (full lines) or 100 nM of BSA (dotted lines) and a flow rate of 30 µl/min (surface: CM5). (D) GSTM1 association and dissociation kinetics of AMPK 221WT at different concentrations (dashed black lines) and a flow rate of 30 µl/min (surface: CM5), single exponential fit of experimental data (grey lines) and corresponding residuals (to assess the quality of the fit, lower panel). Representative sensorgrams of at least two repetitions are shown. Bars on the top of sensorgrams indicate protein injection (association, black) or injection of running buffer (white).

To obtain quantitative data on the GST/AMPK interaction in respect to kinetics and affinity, we performed a series of *in vitro* experiments with surface plasmon resonance spectroscopy (SPR). The GST interaction partner was chosen for covalent immobilization since it appeared more stable in this setup. We first used a sensor chip where the gold surface had been functionalized with a self assembled monolayer that reduces non-specific adsorption to the surface and allows immobilization of low ligand densities for analyzing GST-Sj. Sensorgrams with AMPK 221WT, 221TD or BSA (negative control) injected onto this surface confirmed a direct GST/AMPK interaction (Fig. 4A) that was not affected by glutathione (not shown). The equilibrium response was 203±41.5 RU for AMPK 221WT, which was reduced to 121±27 RU for AMPK 221TD, as compared to 8±3.7 RU for BSA (Fig. 4B). Conventional CM5 sensor chips were then used to confirm a specific interaction of rat GSTM1 or GSTP1 with rat AMPK (Fig. 4C) and to extract affinity data for GSTM1 by injecting an AMPK concentration series (Fig. 4D). The simple kinetics could be very well fitted to a Langmuir 1∶1 model (Fig. 4D) as seen by the very low residuals of the fit (<1 RU). The fast association (k_a_ = 3,1·10^5^ M^−1^ s^−1^) and the very slow dissociation (k_d_ = 1,6·10^−3^ s^−1^) resulting in a K_D_ of about 5 nM.

### GST/AMPK complexes do not lead to relevant GST phosphorylation but increase GST activity

**Figure 5:**
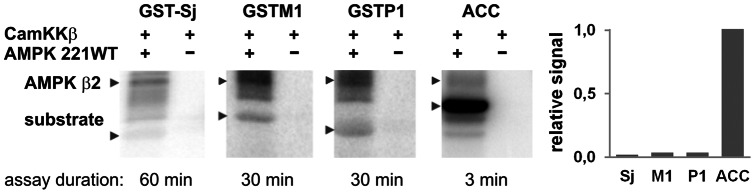
GST is a poor AMPK substrate. AMPK 221WT (4 pmol) pre-activated by CamKKβ (1 pmol) does not phosphorylate GST-Sj and phosphorylates GSTM1 and -P1 only at low levels as compared to ACC (all at 200 pmol). *In vitro* phosphorylation assays were run for 3 min (GST-ACC), 30 min (GSTP1, GSTM1) or 60 min (GST-Sj) and analyzed by SDS-PAGE and Typhoon phosphoimager. Note the autophosphorylation of the AMPK β-subunit. Representative data with quantification are shown. Detailed phosphorylation kinetics is shown in Fig. S2.

To gain insight into the putative role(s) of GST/AMPK complexes, we first examined whether they lead to GST phosphorylation and/or affect GST activity. *In vitro* phosphorylation assays with CamKKβ-activated AMPK and a 50-fold excess of GSTs revealed no phosphorylation of GST-Sj and slow, very low level phosphorylation of GSTM1 and GSTP1 as compared to ACC (Fig. 5), reaching less than 7% of the ACC phosphorylation level within 1 hour (Fig. S2). Presence of glutathione did not further increase this phosphorylation (not shown) as it was reported for PKA and PKC [43], and no specific phosphosites could be identified by mass spectrometry (not shown). However, in an activity assay using the model substrate 1-chloro-2,4-dinitrobenzene (CDNB), addition of AMPK 221WT to GSTM1 or GSTP1 led to a moderate increase of *v_max_* by about 25% at almost unchanged apparent *K_m_* (Table 1, Fig. S3). This increase occurred only after mixing GST with AMPK, not with BSA, and did not require addition of active AMPK 221TD (Table 1). Thus, GST activation is not due to unspecific stabilization by protein addition and unrelated to the faint and slow GST phosphorylation. Rather, specific GST/AMPK complex formation itself altered the catalytic properties of GST, since GST activation also depended on the amount of AMPK 221WT added (Fig. S3).

**Table 1:** Enzyme kinetic parameters of GSTP1 in presence or absence of AMPK or BSA.

	*v_max_*	*k_cat_*	*K_m (CDNB)_*
	*(U mg^−1^)*	*(s^−1^)*	*(mM)*
GSTM1	21,6±0.8	16,9±0,5	0,037±0.005
GSTM1 + BSA	20,9±0.8	16,4±0,5	0,033±0.006
GSTM1 + AMPK 221	25,5±0.7	20,0±0,5	0,045±0,005
GSTM1 + AMPK 221TD	25,9±0,9	20,3±0,6	0,043±0,006
GSTP1	20.4±2.9	16,0±2,3	1.9±0.4
GSTP1 + AMPK 221	24.6±1.1	19,3±0,9	1.5±0.1
GSTP1 + AMPK 221-P ^1^)	26,8±0.5	21,0±0,8	1,7±0.1

GST enzyme activity was determined with variable concentrations of model substrate 1-chloro-2,4-dinitrobenzene at a fixed glutathione concentration (10 mM for GSTM1, 2 mM for GSTP1) at 25°C. V_max_ and K_m_ values were obtained by direct fitting of values to Michaelis-Menten kinetics. Enzyme activity given in U is equivalent to µmol/min. Values are means ± SD, n = 3. ^1)^ AMPK221 pre-activated by phosphorylation with CamKKβ.

### GST/AMPK complexes lead to AMPK glutathionylation and activation

**Figure 6:**
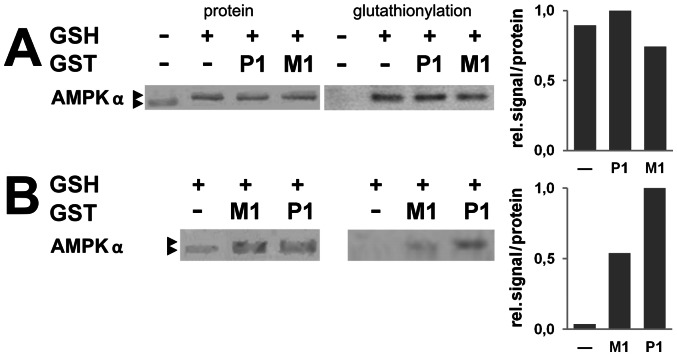
Glutathionylation of AMPK is facilitated by GST. Glutathionylation assays were performed (A) with AMPK 221WT (1 μM) in absence or presence of GSTM1 or -P1 (0,5 μM) and 10 mM glutathione or (B) with AMPK 221WT (1 μM, additionally pre-reduced with ß-mercaptoethanol) in absence or presence of GSTM1 or -P1 (10 μM) and 0,1 mM glutathione. AMPK modification was detected either as a molecular mass shift of GST protein in SDS-PAGE (see arrows in Ponceau protein stain, “protein”) or by direct detection of glutathione by immunoblotting (“glutathionylation”). Note: As soon as glutathione is present, AMPK is almost quantitatively glutathionylated in (A), while additional presence of GST is needed for glutathionylation in (B). Left: representative data; right: quantification (mean, n = 2).

It has previously been shown that GSTM1 and -P1 were able to modulate signal transduction through an interaction with JNK and/or other stress activated kinases ([40], [44], reviewed in [39]). Hence, we hypothesized that GST could modulate AMPK activity by glutathionylation as it was shown recently [20]. At very high glutathione concentrations of 10 mM, spontaneous auto-glutathionylation occurred with AMPK 221WT already in absence of mammalian GSTs (Fig. 6A). However, at more limiting conditions using 0,1 mM glutathione and AMPK pre-reduced with β-mercaptoethanol, auto-glutathionylation was almost absent (Fig. 6B). In this case, presence of GSTM1 and -P1 increased glutathionylation of AMPK to immunodetectable levels, also visible as a partial shift of AMPK to a higher molecular mass (Fig. 6B).

**Figure 7:**
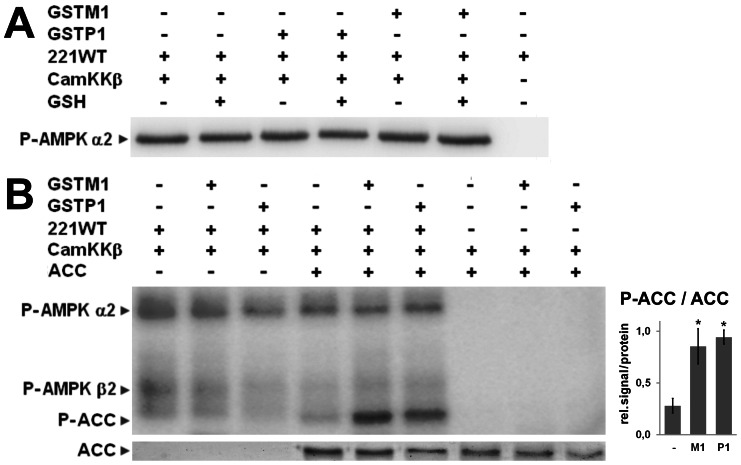
AMPK glutathionylation does not affect its phosphorylation by CamKKβ, but increases phosphorylation of downstream substrate. (A) GSTM1 or -P1 (62,5 pmol) were pre-incubated with AMPK 221WT (12.5 pmol) with or without 10 mM glutathione, in presence of ATP prior to addition of CamKKβ (0.63 pmol). Phosphorylation assays were subjected to immunoblot analysis using anti-P-T172-α AMPK antibody. (B) AMPK 221WT pre-activated with CamKKβ in kinase buffer with cold ATP and glutathionylated with 0,1 mM glutathione in presence or absence of GSTM1 or -P1, both as described above and in Fig. 6, were incubated with ACC (200 pmol) and [γ-^32^P]ATP. *In vitro* phosphorylation assays were analyzed by SDS-PAGE, Ponceau protein staining (lower panel) and Typhoon phosphoimager (upper panel) are shown. Left: representative data; right: quantification of lanes in presence of glutathione (mean ± SD, n = 4; * = p<0,01 *versus* no GST). A control experiment lacking CamKKβ is shown in Fig. S4. Note: AMPK autophosphorylation of α- and β-subunits.

We then analyzed the effect of AMPK S-glutathionylation for AMPK signaling. *In vitro* phosphorylation of AMPK 221WT at αT172 by its upstream kinase CamKKβ was identical in presence or absence of glutathione, and also not further modified by GSTM1 or -P1 (Fig. 7A). However, phosphorylation of the AMPK downstream substrate ACC was clearly increased with AMPK 221WT preparations that had been glutathionylated before in presence of glutathione by GSTM1 and -P1 as above, compared to controls lacking mammalian GSTs (Fig. 7B). Activation of AMPK by CamKKβ was a prerequisite for this ACC phosphorylation. CamKKβ (Fig. 7B) and AMPK alone (Fig. S4) or combined with GSTs did not affect ACC phosphorylation. These results suggest that GST-dependent S-glutathionylation of AMPK *in vitro* indeed increases kinase activity in the same way as previously shown for H_2_O_2_-dependent AMPK glutathionylation [20].

## Discussion

The energy stress sensor AMPK can be activated by ROS and RNS, possibly *via* different AMP-dependent and -independent mechanisms [12]–[14] and is involved in cellular redox regulation [16] and antioxidative defense via induced expression of various antioxidative pathways [17]–[19]. Exposure of AMPK to the strong oxidant hydrogen peroxide at high glutathione concentrations induces non-enzymatic S-glutathionylation of AMPK α- and β- subunits which in turn activates the kinase [20]. Our study adds another element to such redox regulation: activation of AMPK via GST-facilitated glutathionylation in the absence of exogenous oxidant that may be relevant to normal physiological conditions. We provide evidence that mammalian GSTM1 and -P1 can rapidly interact with AMPK, become enzymatically activated by this interaction, and assist in turn in glutathionylation and activation of AMPK as we show *in vitro*.

It has previously been demonstrated that GSTM1 and -P1 were able to modulate signal transduction through interactions with JNK and/or other stress activated kinases ([40], [44], reviewed in [39]), and that this can involve GST phosphorylation or modification of the interacting kinase [40]–[44]. However, interaction with AMPK led only to slow and low-level phosphorylation of GSTM1 and -P1; its importance (if any) remains to be elucidated. By contrast, complex formation alone was sufficient to increase activity of bound GST and, importantly, to glutathionylate and activate AMPK under *in vitro* conditions where auto-glutathionylation is low, i.e. in the absence of strong oxidants. It is worth noting that protein interaction partners of GST were mostly also identified as targets for S-glutathionylation (e.g. [29], [31], [55]). The residues glutathionylated within AMPK, α-Cys299 and α-Cys304 [20], activate AMPK rather due to direct conformational changes as those produced by allosteric AMP regulation. AMPK glutathionylation did not make AMPK a better substrate for the upstream kinase CamKKβ, at least with the recombinant enzymes in the reconstituted *in vitro* system applied here.

Reversible protein modification by cysteine glutathionylation is increasingly recognized as an important signaling mechanism by which cells can respond effectively and reversibly to redox inputs [21], [23]. S-glutathionylation inhibits or activates a number of protein kinases (PKA, PKC, MEKK1, ASK1) and phosphatases (reviewed in [23]). Although the understanding of the S-glutathionylation cycle is still limited, there is evidence for the participation glutaredoxins and GST isoforms, including GSTM1 and -P1 [28], [31], [35]. GSTP1P2 knockout mice and cells expressing dead mutants of GSTP have a diminished capacity to S-glutathionylate proteins [28]. GSTP plays an essential role in the S-glutathionylation of 1-cys peroxiredoxin [29], [30], [56]. Even more, a glutathionylation cycle was recently described to regulate aldose reductase [31]. Here, sequential glutathiolyation/deglutathioylation is catalyzed by GSTP and GRx *in vitro* and *in vivo*, correlated with physical association of the reductase with either GSTP or GRx [31]. Since the GST catalytic activity is necessary for lowering the pKa of the glutathione cysteine thiol [45], altered catalytic properties as we observed with GSTM1 and -P1 in complex with AMPK may be relevant for the GST glutathionylation function.

These findings fit very well into the emerging role of AMPK as a redox switch in oxidative stress and redox signaling. AMPK is activated by ROS/RNS via different mechanisms: impaired mitochondrial ATP generation will translate into increased cytosolic AMP/ATP and ADP/ATP ratios that activate AMPK [12], but ROS/RNS may also directly affect upstream mediators and kinases [13], [14] or AMPK itself by glutathionylation [20]. Activated AMPK in turn up-regulates the cellular antioxidative defense machinery, mainly via the FOXO3 transcription factor: manganese superoxide dismutase [17]–[19], catalase [17], [18], thioredoxin [17], [57], metallothioneins [58], or uncoupling protein 2 [15]. Among AMPK-FOXO3-induced genes are also γ-glutamylcysteine synthase, the first enzyme in glutathione biosynthesis [17], glutathione peroxidase [18] that uses glutathione to reduce lipid and hydrogen peroxides, as well as GSTM1 [58].

The interaction leading to GST/AMPK complexes seems to be rather specific for the GST-Mu and -Pi families, since it was not observed with GST-Alpha and -Omega isoforms (not shown). It does not involve the AMPK α-subunit, as we have recently reported for fumarate hydratase [59], but the β-subunit, as also seen with several other putative mammalian AMPK interactors (IntAct database, [60]) and the yeast and plants orthologs [61], [62]. Our data suggest interaction with the very N-terminal part of the β-subunit, a domain that is fairly well conserved across the AMPK protein family but lacks in the solved core structures of mammalian AMPK and its yeast ortholog ([10]; reviewed in [63]), possibly due to its high flexibility. The physical interaction of AMPK with GST-Sj calls for a note of caution for using GST fusion proteins in pull-down assays to identify AMPK interaction partners.

GST-mediated glutathionylation and activation of AMPK may be considered a possible additional layer of AMPK regulation linking the energy-stress sensor to redox regulation and anti-oxidative defense. Our present data are novel in that they provide a mechanism for glutathionylation-dependent AMPK activation at low oxidative capacity, as compared to the highly oxidative conditions used in an earlier study [20] which may not mimic peroxide concentrations generated intracellularly [22], [26]. Further studies have to show the specific importance of this mechanism for *in vivo* regulation of AMPK activity.

## Supporting Information

Figure S1 **The Strep-tag in Strep-GST constructs is phosphorylated by AMPK.** Phosphorylation of GSTP1(200 pmol) and GSTM1 (40 pmol) in Strep-tagged (P1, M1) and Strep-tag-free forms (P1c, M1c) by AMPK221 (4 pmol) activated by CamKKβ (1 pmol). *In vitro* phosphorylation for 10 min at 37°C was analyzed by SDS-PAGE and Typhoon phosphoimager (top panel) and control Coomassie stain for protein loading (bottom panel). Control lanes lack AMPK221 but contain CamKKβ.
(PDF)

Figure S2 **Substoichiometric phosphorylation of GSTP1 by AMPK** ***in vitro*** **.** (A) Phosphorylation time course of GSTP1 or ACC (200 pmol each) by AMPK221 (4 pmol) activated by CamKKβ (1 pmol). *In vitro* phosphorylation for 5 to 60 min at 37°C was analyzed by SDS-PAGE and Typhoon phosphoimager. Control lanes lack AMPK221 but contain CamKKβ. (B) Quantification of (A) using Image Quant TL, using normalization to maximal ACC phosphorylation and fitting to phosphorylation enzyme kinetics.
(PDF)

Figure S3 **GSTM1 and -P1 are activated in complexes with AMPK** ***in vitro*** **.** Enzyme activity of or 20 μg GSTM1 (A) or 30 μg GSTP1 (B) in absence or presence of 5 or 15 µg AMPK221WT at different concentrations of the model substrate CDNB and saturating glutathione concentrations.
(PDF)

Figure S4 **Increased phosphorylation of AMPK downstream substrate depends on the presence of AMPK-activating upstream kinase CamKKβ.** AMPK 221WT preactivated with CamKKβ in kinase buffer with cold ATP and glutathionylated with 0,1 mM glutathione in presence or absence of GSTM1 or -P1, both as described in Figs. 6 and 7, were incubated with ACC (200 pmol) and [γ-^32^P]ATP. *In vitro* phosphorylation assays were analyzed by SDS-PAGE, Ponceau protein staining (lower panel) and Typhoon phosphoimager (upper panel) are shown. Note: AMPK autophosphorylation in particular of the α-subunit.
(PDF)
