# Supplementary material for: Glutathione S-Transferases Interact with AMP-Activated Protein Kinase: Evidence for S-Glutathionylation and Activation In Vitro
Source: PLoS One. 2013 May 31;8(5):e62497. doi: 10.1371/journal.pone.0062497 (PMC3669356; doi:10.1371/journal.pone.0062497)
Supplement: Figure S1 — The Strep-tag in Strep-GST constructs is phosphorylated by AMPK. Phosphorylation of GSTP1(200 pmol) and GSTM1 (40 pmol) in Strep-tagged (P1, M1) and Strep-tag-free forms (P1c, M1c) by AMPK221 (4 pmol) activated by CamKKβ (1 pmol). In vitro phosphorylation for 10 min at 37°C was analyzed by SDS-PAGE and Typhoon phosphoimager (top panel) and control Coomassie stain for protein loading (bottom panel). Control lanes lack AMPK221 but contain CamKKβ. (PDF) [file pone.0062497.s001.pdf]

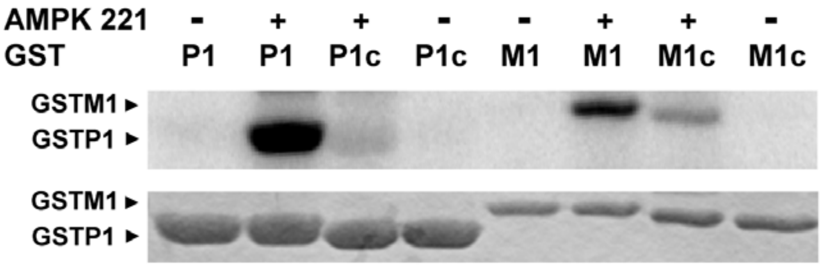

Figure S1. The Strep-tag in Strep-GST constructs is phosphorylated by AMPK. Phosphorylation of GSTP1(200 pmol) and GSTM1 (40 pmol) in Strep-tagged (P1,M1) and Strep-tag-free forms (P1c, M1c) by AMPK221 (4 pmol) activated by CamKKβ (1 pmol). *In vitro* phosphorylation for 10 min at 37°C was analyzed by SDS-PAGE and Typhoon phosphoimager (top panel) and control Coomassie stain for protein loading (bottom panel). Control lanes lack AMPK221 but contain CamKKβ.
