# Supplementary material for: Glutathione S-Transferases Interact with AMP-Activated Protein Kinase: Evidence for S-Glutathionylation and Activation In Vitro
Source: PLoS One. 2013 May 31;8(5):e62497. doi: 10.1371/journal.pone.0062497 (PMC3669356; doi:10.1371/journal.pone.0062497)
Supplement: Figure S2 — Substoichiometric phosphorylation of GSTP1 by AMPK in vitro . (A) Phosphorylation time course of GSTP1 or ACC (200 pmol each) by AMPK221 (4 pmol) activated by CamKKβ (1 pmol). In vitro phosphorylation for 5 to 60 min at 37°C was analyzed by SDS-PAGE and Typhoon phosphoimager. Control lanes lack AMPK221 but contain CamKKβ. (B) Quantification of (A) using Image Quant TL, using normalization to maximal ACC phosphorylation and fitting to phosphorylation enzyme kinetics. (PDF) [file pone.0062497.s002.pdf]

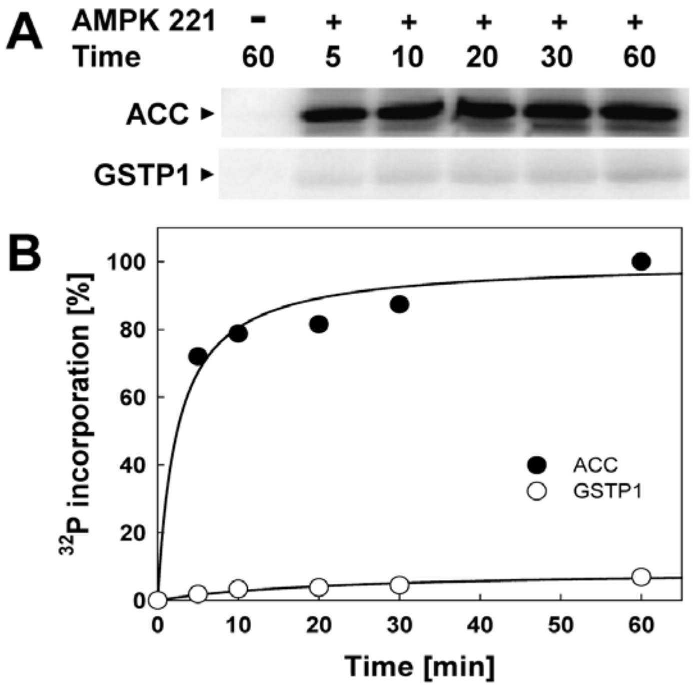

Figure S2. Substoichiometric phosphorylation of GSTP1 by AMPK *in vitro*. (A) Phosphorylation time course of GSTP1 or ACC (200 pmol each) by AMPK221 (4 pmol) activated by CamKKβ (1 pmol). *In vitro* phosphorylation for 5 to 60 min at 37°C was analyzed by SDS-PAGE and Typhoon phosphoimager. Control lanes lack AMPK221 but contain CamKKβ. (B) Quantification of (A) using Image Quant TL, using normalization to maximal ACC phosphorylation and fitting to phosphorylation enzyme kinetics.
