# Supplementary material for: Glutathione S-Transferases Interact with AMP-Activated Protein Kinase: Evidence for S-Glutathionylation and Activation In Vitro
Source: PLoS One. 2013 May 31;8(5):e62497. doi: 10.1371/journal.pone.0062497 (PMC3669356; doi:10.1371/journal.pone.0062497)
Supplement: Figure S3 — GSTM1 and -P1 are activated in complexes with AMPK in vitro . Enzyme activity of or 20 μg GSTM1 (A) or 30 μg GSTP1 (B) in absence or presence of 5 or 15 µg AMPK221WT at different concentrations of the model substrate CDNB and saturating glutathione concentrations. (PDF) [file pone.0062497.s003.pdf]

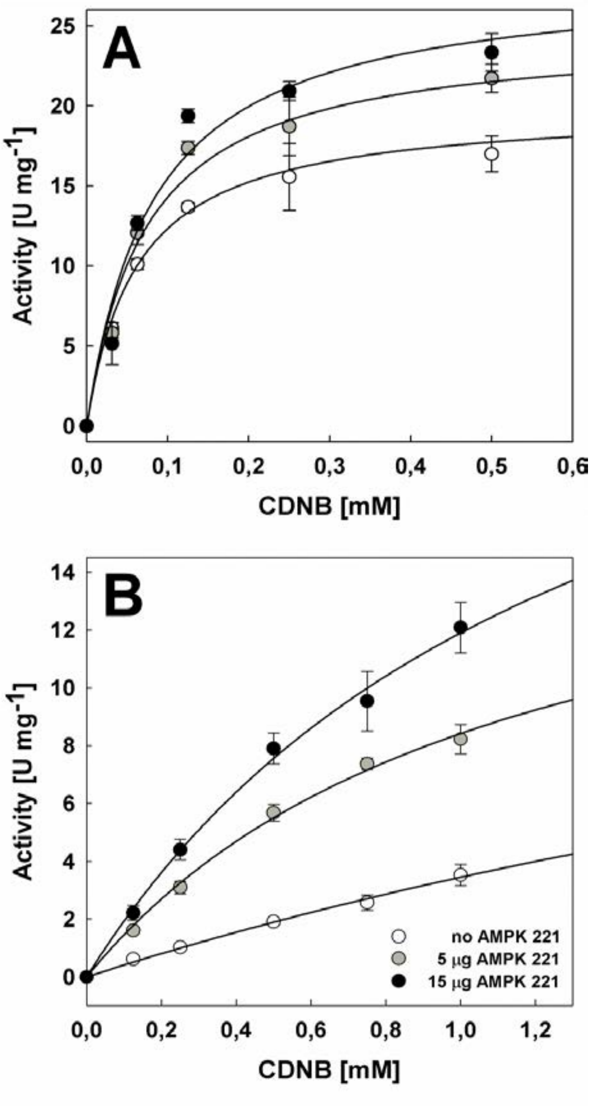

Figure S3. GSTM1 and -P1 are activated in complexes with AMPK *in vitro*. Enzyme activity of or 20 µg GSTM1 (A) or 30 µg GSTP1 (B) in absence or presence of 5 or 15 µg AMPK221WT at different concentrations of the model substrate CDNB and saturating glutathione concentrations.
