# Supplementary material for: Glutathione S-Transferases Interact with AMP-Activated Protein Kinase: Evidence for S-Glutathionylation and Activation In Vitro
Source: PLoS One. 2013 May 31;8(5):e62497. doi: 10.1371/journal.pone.0062497 (PMC3669356; doi:10.1371/journal.pone.0062497)
Supplement: Figure S4 — Increased phosphorylation of AMPK downstream substrate depends on the presence of AMPK-activating upstream kinase CamKKβ. AMPK 221WT preactivated with CamKKβ in kinase buffer with cold ATP and glutathionylated with 0,1 mM glutathione in presence or absence of GSTM1 or -P1, both as described in Figs. 6 and 7, were incubated with ACC (200 pmol) and [γ-32P]ATP. In vitro phosphorylation assays were analyzed by SDS-PAGE, Ponceau protein staining (lower panel) and Typhoon phosphoimager (upper panel) are shown. Note: AMPK autophosphorylation in particular of the α-subunit. (PDF) [file pone.0062497.s004.pdf]

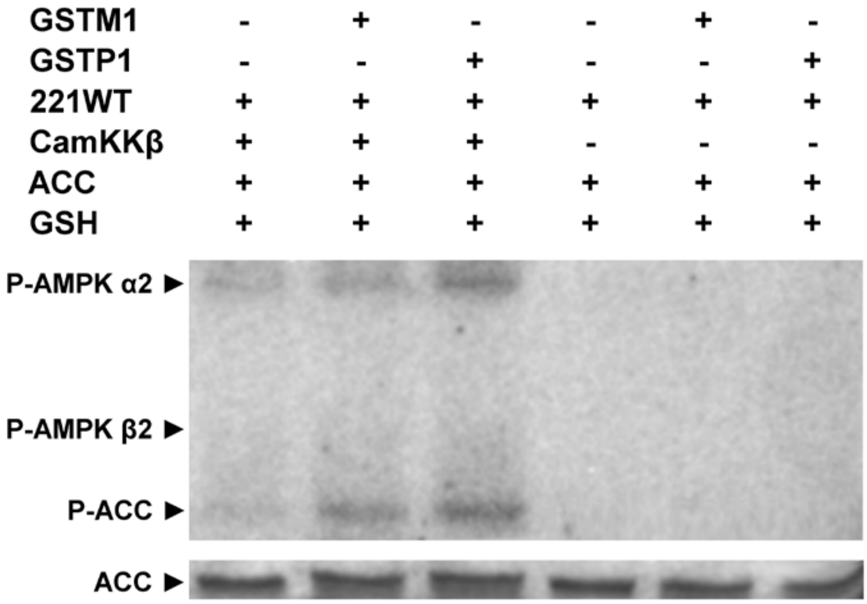

Figure S4. Increased phosphorylation of AMPK downstream substrate depends on the presence of AMPK-activating upstream kinase CamKKβ. AMPK 221WT preactivated with CamKKβ in kinase buffer with cold ATP and glutathionylated with 0,1 mM glutathione in presence or absence of GSTM1 or -P1, both as described in Figs 6 and 7, were incubated with ACC (200 pmol) and [γ-^{32}P]ATP. *In vitro* phosphorylation assays were analyzed by SDS-PAGE, Ponceau protein staining (lower panel) and Typhoon phosphoimager (upper panel) are shown. Note: AMPK autophosphorylation in particular of the α-subunit.
